# The Mortality of the Tuberculous

**Published:** 1913-04-19

**Authors:** 


### THE MORTALITY OF THE TUBERCULOUS.

The Galton Eugenics Laboratory has made another attempt to elucidate various points connected with the future of consumptives. This time the data are derived from American sources chiefly, but also from two Scotch sanatoriums, while some sort of "control" is obtained from the case-books of a mid-nineteenth century American tuberculosis specialist, the late Dr. Austin Flint. A rather heterogeneous material therefore; but since the subject, as Messrs. Elderton and Perry, the authors of the memoir, rightly say, is of much topical importance, their findings deserve attention. More especially are they noteworthy, we would add, on account of the caution evident in formulating them. "Possible weaknesses in the material" are fully mentioned, and the notorious dogmatism of the mathematician avoided. Dr. Flint, it is owned, may not have contemplated any statistical use of his records, and have even noted the cases which lived a long time, rather than those which died early. Although the mortality of those more recently admitted to the Adirondack Sanatorium is considerably lighter than that of the earlier admissions, a large part of this decrease in mortality is due to a different selection and possibly a different classification, at any rate on discharge. But if one begins to think—"The sort of verdict to be expected from a eugenist!"—the suspicion of *parti pris* is dispelled by the next sentences, to the effect that "the greater success in selecting suitable cases . . . implies a real advance in knowledge. Such knowledge might become a matter of considerable social importance."

With favourable impressions, then, one turns to the conclusions, conveniently stated at the end of the paper. They are strictly limited as to scope. The mortality of patients with T.B. in the sputum is more than twice as heavy as that of the rest. And lest it be said that the latter were non-tuberculous, it is added that their mortality again is considerably heavier than that of the English Life Table. In this connection there may be recalled a finding in a recent paper of Dr. Bardswell's, that patients who lost their bacilli under treatment did much better than those who kept them. Unambitious little determinations like these, which do no more than confirm fairly authoritatively the traditions of the modern treatment of phthisis, are often of the greatest immediate practical significance. Conclusion number four is another one of this kind, that consumption in the parents does not make the patient's own outlook any worse. As to tuberculin, it is stated that there is no evidence here that it lengthens life as compared with ordinary sanatorium treatment. The mode of treatment used in the cases on which this finding is based was that of Denys. The professors of other forms of tuberculin therapy will doubtless feel that their withers are unwrung—perhaps justly, although it might be said that, as Denys' course of treatment is usually a very long one, some unfavourable cases may have been excluded. And if widespread ambulant tuberculin treatment, such as is now coming in, is to be appraised, the comparison will have to be between tuberculin treated cases and those who get no chance of a stay at a sanatorium. But figures for this will be unavailable for a long time yet, and not very accessible ever. However, a central authority for directing tuberculosis research, although it has obvious dangers to avoid in the way of hampering individual initiative, may have also the advantage of a rich store of statistics upon which actuarial investigations like the present one may be more solidly based.

